# Introduction to the Special Issue “Personality and Individual Differences”

**DOI:** 10.3390/jintelligence12080078

**Published:** 2024-08-08

**Authors:** Kay Brauer, René T. Proyer

**Affiliations:** Department of Psychology, Germany Martin Luther University Halle-Wittenberg, 06099 Halle, Germany; rene.proyer@psych.uni-halle.de

## 1. Introduction

The study of intelligence is one of the foundations of scientific psychology, and for more than a century, researchers from psychology and other disciplines such as neuroscience, genetics, and education have been interested in extending the knowledge about the structure and correlates of intelligence. While there is yet no consensus regarding one final structural model of intelligence as models differ with regard to their dimensionality, hierarchy, and distinctiveness of factors, there is robust evidence that available tests of cognitive abilities allow for the reliable and valid assessment of intra- and interindividual expressions of intelligence. We are happy to introduce the Special Issue entitled “Personality and Individual Differences” in the *Journal of Intelligence*, which has the aim to extend the knowledge about the interplay of personality traits and cognitive abilities and to provide research on the intersection between intelligence and individual differences. 

## 2. Personality and Intelligence—Two Separate Worlds?

Historically, intelligence was localized and studied in the field of individual differences and personality by paragons such as William Stern and Raymond B. Cattell, who worked on both the study of temperament and cognitive abilities. However, it appears that different research traditions exist in which they were either studied together or divided into subfields. In the tradition of German-speaking countries, they were usually studied together. Take, for example, this description of what constitutes “personality” from Franziska Baumgarten, a paragon of the lexical approach to personality research: “[…] we define personality as the totality of all psychophysical functions and qualities integrated into a whole and active within a human being, and we distinguish various constitutive elements (constituents [orig. *Konstituenten*]) within it:

1. Sensory perceptions, thoughts along with the functions that receive, process, and store them (attention and memory).
2. Feelings and affect/emotions.
3. Drives, instincts, and propensities.
4. Character traits.
5. Intelligence.
6. Temperament.
7. Specific aptitudes and talents” (Baumgarten 1933, p. 16; translated by the authors).

It is also striking that research on the intersection between personality traits and intelligence has been comparatively rare in the flagship journals of intelligence. This notion is supported by bibliometric analyses of the two major outlets of the field of intelligence (Parra-Martinez et al. 2023; Pesta 2018; Pesta et al. 2018; Wicherts 2009): The *Journal of Intelligence* (founded in 2013) and *Intelligence* (founded in 1977). The quoted bibliometric analyses show that the study of personality in relation to intelligence plays only a comparatively minor role. For example, in Wicherts’ (2009) analysis of keywords and topics from studies published in *Intelligence*, the terms “personality” or “traits” are *not* mentioned in the literature overview. Similarly, Pesta and colleagues’ (2018) analysis of articles published between 2000 and 2016 in this outlet showed that personality appeared more frequently as a keyword in articles but yielded only rank 35. Interestingly, Pesta (2018) updated these analyses and, additionally, also examined the sources of citations. They found that *Personality and Individual Differences*, along with the journal *Learning and Individual Differences*, generated the most citations of articles from *Intelligence* in 2016 (about 10% of all citations).

The analysis of the citation rates over time suggests that the field of personality and individual differences remains concerned with the relationship and interaction of personality and intelligence and cognitive abilities. This is not surprising, considering that there is an increasing interest in understanding individual differences variables not only from the perspective of emotional experiences but also implementing views on cognitive abilities; for example, intelligence has been added as an important component for the accurate interpersonal perception of personality traits (e.g., Murphy and Hall 2011), its interplay with trait openness (Zhang and Ziegler 2015), or the role of (in)accurate self-perceptions of intelligence (Hofer et al. 2022). This is particularly well reflected in publications from the *Journal of Intelligence*. Parra-Martinez and colleagues (2023) provided the first bibliometric analysis for this journal and noted that there is an increasing trend in studies addressing the interplay with personality since 2016. This is also reflected in the increasing occurrence of “personality” as a keyword since 2019, yet the topic is still comparatively peripheral. Capitalizing on the increasing interest in the interplay between cognitive and non-cognitive individual differences (see also Colom et al. 2019), we hope that our Special Issue contributes to stimulating research on intelligence under the lens of personality and non-cognitive individual differences variables and vice versa and that the consideration of both extends our understanding of intra- and interpersonal psychological phenomena.

## 3. An Overview of the Special Issue

We are grateful to the authors who contributed their high-quality work to this Special Issue, which includes research from Austria, Germany, Canada, China, and the U.S.A. These studies employ diverse methods to learn more about various indicators of cognitive abilities and personality traits.

Biesok et al. (2024) examine figurative language comprehension in relation to cognitive abilities as defined by the Cattell–Horn–Carroll model. Using the data of 909 participants, they tested how cognitive abilities contribute to understanding metaphorical and symbolical meanings conveyed through figurative language. The study shows that comprehension of figurative language is related to but not redundant with fluid intelligence and *g*, as their analyses of potential measurement models and associations show. At the same time, their data show that understanding figurative language relates to the personality trait of openness and its facets, intellectual curiosity, and aesthetic sensitivity. Thus, showing the complexity of the parallel roles of cognitive and non-cognitive traits for the understanding of figurative language as a phenomenon that guides everyday communications.

Grinschgl et al. (2023) conducted a pre-registered study investigating the factors relating to the openness of individuals to engage in cognitive enhancement (i.e., *cognitive enhancement*). They presented 257 participants with eight possible types of active and passive enhancement strategies through vignettes (e.g., pharmacological, game-based, and neurofeedback) and collected self-report data of broader (big five traits) and narrower traits (e.g., narcissism), implicit theories of intelligence, and subjective and psychometric measures of intelligence. Self-estimates and psychometrically measured, as well as theories about intelligence, did not relate to openness to use cognitive enhancement. However, higher interest in science fiction and greater openness were associated with increased willingness to enhance cognitive abilities. Their findings are one of the first to examine the role of interindividual differences for the willingness to accept and engage in enhancing one’s cognitive abilities through active and passive strategies.

Lau et al. (2023) studied the associations between cognitive abilities and both normative traits (i.e., big five traits) and maladaptive (personality pathology) personality traits. They analyzed data from 201 outpatients who completed verbal and reasoning tests and measures of personality and psychopathology. Using profile analyses, they found that personality profiles (based on the big five traits, the maladaptive traits, or their combination) did not relate to cognitive abilities. Thus, replicating and extending evidence on the orthogonality of personality traits and cognitive abilities.

Li et al. (2024) utilized longitudinal data with a 1-year interval from 509 adolescents and examined the associations between working memory and critical thinking (i.e., weighing available evidence with regard to evaluating information as true or false) measured as ability and as a disposition. In short, their findings from longitudinal path analyses considering all data simultaneously show that all measures are characterized by stability over time and that critical thinking as ability is predicted by working memory, whereas critical thinking by means of a stable disposition is predicted by critical thinking ability but unrelated to working memory over time. Li et al.’s findings add to the knowledge about how working memory and inclinations to weigh information as a disposition relate to the ability of thinking critically.

Ma et al. (2024) examined the association between reasoning performance and media multitasking under the lens of personality traits and family-based socioeconomic status (SES). Their study is based on a sample of 777 university students completing ability measures of reasoning and several self-report questionnaires to examine personality traits such as the big five traits and impulsiveness. After showing that greater media multitasking relates to lower reasoning performance, Ma et al. found that family SES and the traits of openness, extraversion, and conscientiousness moderated the associations. Hence, Ma et al. expanded on the role of both personality and intelligence in relation to individual differences in an everyday behavior such as media multitasking, showing that, for example, extraverts are used to receiving greater input from multiple sources, whereas introverts are more affected by dealing with multiple sources of media simultaneously and dealing with an overflow of information and its effects on attention.

Finally, Willoughby et al. (2023) tested the relations between the big five personality traits on the level of domains and facets with cognitive abilities. Using data from 481 and assessing cognitive abilities with tests such as matrix reasoning and 3D rotations, they also employed a measure assessing reaction times (RT) in the sense of Jensen’s approach to intelligence. Their findings showed again that the personality domains are unrelated to RT-based indicators of cognitive abilities, except for minor yet stable associations with openness/intellect. Also, they show that scores of intellect have incremental value beyond psychometrically measured intelligence when predicting RT. Willoughby et al. discuss the integration and consideration of cognitive aspects when describing individual differences in personality traits, especially when models and tests of personality include indicators of curiosity and intellect.

The articles presented in this Special Issue cover a diverse range of topics from the field of intelligence, personality, and individual differences that will contribute to the knowledge in the field. We hope that the important and interesting findings from these contributions highlight and stimulate research on the intersection and interplay between broad and narrow non-cognitive and cognitive individual differences variables, as the studies from this Special Issue illustrate the merits of considering both components. We expect that the interplay will help to inform and test established and new theories, be it regarding cognitive aspects in perceptions of situations or the role of non-cognitive traits for understanding how people use their cognitive abilities in different contexts.

## References

[B1-jintelligence-12-00078] Baumgarten Franziska (1933). Die Charaktereigenschaften [Charater Traits]. Francke.

[B2-jintelligence-12-00078] Biesok Andra, Ziegler Matthias, Montag Christiane, Nenchev Ivan (2024). Adding a piece to the puzzle? The allocation of figurative language comprehension into the CHC model of cognitive abilities. Journal of Intelligence.

[B3-jintelligence-12-00078] Colom Roberto, Bensch Doreen, Horstmann Kai. T., Wehner Caroline, Ziegler Matthias (2019). Special issue “the ability–personality integration”. Journal of Intelligence.

[B4-jintelligence-12-00078] Grinschgl Sandra, Berdnik Anna-Lena, Stehling Elisabeth, Hofer Gabriela, Neubauer Aljoscha C. (2023). Who wants to enhance their cognitive abilities? Potential predictors of the acceptance of cognitive enhancement. Journal of Intelligence.

[B5-jintelligence-12-00078] Hofer Gabriela, Mraulak Valentina, Grinschgl Sandra, Neubauer Aljoscha C. (2022). Less-intelligent and unaware? Accuracy and Dunning–Kruger effects for self-estimates of different aspects of intelligence. Journal of Intelligence.

[B6-jintelligence-12-00078] Lau Chloe, Bagby R. Michael, Pollock Bruce G., Quilty Lena (2023). Five-Factor Model and DSM-5 Alternative Model of Personality Disorder profile construction: Associations with cognitive ability and clinical symptoms. Journal of Intelligence.

[B7-jintelligence-12-00078] Li Shuangshuang, Wang Ziyue, Sun Yijia (2024). Relationship between thinking dispositions, working memory, and critical thinking ability in adolescents: A longitudinal cross-lagged analysis. Journal of Intelligence.

[B8-jintelligence-12-00078] Ma Yuning, Yin Jinrong, Xuan Hongzhou, Ren Xuezhu, He Jie, Wang Tengfei (2024). Personality traits and family SES moderate the relationship between media multitasking and reasoning performance. Journal of Intelligence.

[B9-jintelligence-12-00078] Murphy Nora A., Hall Judith A. (2011). Intelligence and interpersonal sensitivity: A meta-analysis. Intelligence.

[B10-jintelligence-12-00078] Parra-Martinez Fabio Andres, Desmet Ophélie Allyssa, Wai Jonathan (2023). The evolution of Intelligence: Analysis of the Journal of Intelligence and Intelligence. Journal of Intelligence.

[B11-jintelligence-12-00078] Pesta Bryan J. (2018). Bibliometric analysis across eight years 2008–2015 of Intelligence articles: An updating of Wicherts (2009). Intelligence.

[B12-jintelligence-12-00078] Pesta Bryan J., Fuerst John, Kirkegaard Emil O. W. (2018). Bibliometric keyword analysis across seventeen years (2000–2016) of intelligence articles. Journal of Intelligence.

[B13-jintelligence-12-00078] Wicherts Jelte. M. (2009). The impact of papers published in Intelligence 1977–2007 and an overview of the citation classics. Intelligence.

[B14-jintelligence-12-00078] Willoughby Emily A., Kim Yuri, Lee James J., DeYoung Colin G. (2023). The construct validity of intellect and openness as distinct aspects of personality through differential associations with reaction time. Journal of Intelligence.

[B15-jintelligence-12-00078] Zhang Jing, Ziegler Matthias (2015). Interaction effects between openness and fluid intelligence predicting scholastic performance. Journal of Intelligence.

